# Mercury Is Taken Up Selectively by Cells Involved in Joint, Bone, and Connective Tissue Disorders

**DOI:** 10.3389/fmed.2019.00168

**Published:** 2019-07-19

**Authors:** Roger Pamphlett, Stephen Kum Jew

**Affiliations:** ^1^Discipline of Pathology, Brain and Mind Centre, Sydney Medical School, The University of Sydney, Sydney, NSW, Australia; ^2^Department of Neuropathology, Royal Prince Alfred Hospital, Sydney, NSW, Australia

**Keywords:** mercury, rheumatoid arthritis, osteoarthritis, connective tissue disorder, synovium, cartilage, fibroblast, endothelial cell

## Abstract

**Background:** The causes of most arthropathies, osteoarthritis, and connective tissue disorders remain unknown, but exposure to toxic metals could play a part in their pathogenesis. Human exposure to mercury is common, so to determine whether mercury could be affecting joints, bones, and connective tissues we used a histochemical method to determine the cellular uptake of mercury in mice. Whole neonatal mice were examined since this allowed histological assessment of mercury in joint, bone, and connective tissue cells.

**Materials and Methods:** Pregnant mice were exposed to a non-toxic dose of 0.5 mg/m^3^ of mercury vapor for 4 h a day on gestational days 14–18. Neonates were sacrificed at postnatal day 1, fixed in formalin, and transverse blocks of the body were processed for paraffin embedding. Seven micrometer sections were stained for inorganic mercury using silver nitrate autometallography, either alone or combined with CD44 immunostaining to detect progenitor cells. Control neonates were not exposed to mercury during gestation.

**Results:** Uptake of mercury was marked in synovial cells, articular chondrocytes, and periosteal and tracheal cartilage cells. Mercury was seen in fibroblasts in the dermis, aorta, esophagus and striated muscle, some of which were CD44-positive progenitor cells, and in the endothelial cells of small blood vessels. Mercury was also present in renal tubules and liver periportal cells.

**Conclusions:** Mercury is taken up selectively by cells that are predominantly affected in rheumatoid arthritis and osteoarthritis. In addition, fibroblasts in several organs often involved in multisystem connective tissue disorders take up mercury. Mercury provokes the autoimmune, inflammatory, genetic, and epigenetic changes that have been described in a range of arthropathies and bone and connective tissue disorders. These findings support the hypothesis that mercury exposure could trigger some of these disorders, particularly in people with a genetic susceptibility to autoimmunity.

## Introduction

The initial pathogenic causes of most connective tissue disorders, and of rheumatoid arthritis and osteoarthritis, remain unknown. In many of these disorders an underlying genetic (1) or epigenetic (2, 3) susceptibility to autoimmunity has been postulated, and circulating autoantibodies to nuclear and other targets are common (4). It has been suggested that exposure to environmental toxicants could play a part in the pathogenesis of joint and connective tissue disorders (5). One such toxicant is mercury, where human exposure is widespread and common (6). Mercury and other toxic metals have now been implicated in the pathogenesis of rheumatoid arthritis, osteoarthritis, systemic sclerosis, mixed connective tissue disorder, systemic lupus erythematosus, fibromyalgia, and Sjogren's syndrome (7–12).

Mercury may be the toxic element of particular relevance to joint and connective tissue diseases (2, 4) since it provokes the autoimmune (13), inflammatory (14), and epigenetic (15, 16) changes that have been described in these disorders. Mercury has also been associated with the presence of many of the autoantibodies found in connective tissue diseases (4), especially those to nuclear proteins (17–20). Only some strains of mice produce autoantibodies when exposed to mercury (20, 21), and gender predispositions to mercury toxicity have been described (22). This suggests that genetic and gender-based susceptibilities may be required to produce these mercury-induced autoantibodies (14).

**Figure 1:**
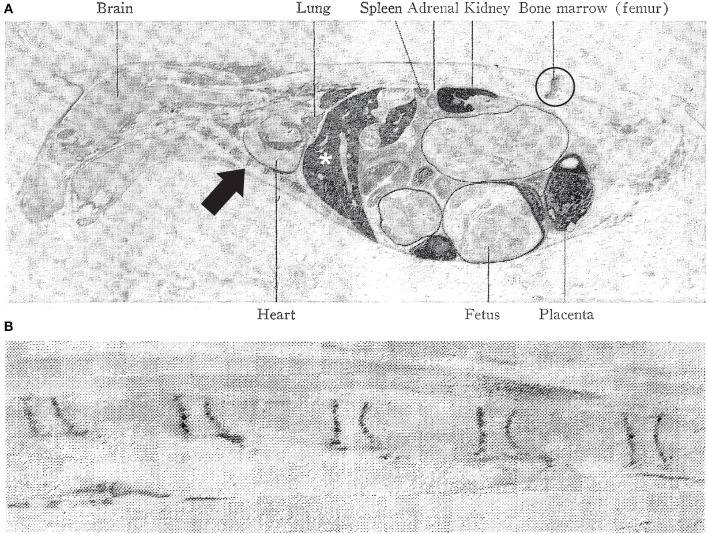
Distribution of mercury in the pregnant mouse. **(A)** Autoradiography of a pregnant mouse 4 days after injection with radioactive mercuric chloride. The liver (asterisk), kidney and placenta contain large amount of dark-appearing mercury. Mercury is present in the hip joint (circled), labeled as “bone marrow (femur)” and at the bone-cartilage junctions in the sternum (e.g., arrow), anterior to the heart. Smaller amounts of mercury are seen in the fetuses. **(B)** Autoradiography of a pregnant mouse vertebral column 16 days after radioactive mercuric chloride injection. Mercury (dark lines) is seen at the junctions between cartilage and bone. Reproduced with permission from Berlin et al. (23).

Information about mercury uptake in vertebrate joints, bone and other connective tissues is scanty, due largely to the difficulty of analyzing toxic elements in hard, mineralized tissues such as bone, which requires decalcification before microtomy. However, evidence for mercury uptake in bone and joints comes from autoradiographic studies in pregnant mice, where mercury is seen in the adult mouse in mid-sagittal images at the bone-cartilage junction of the vertebrae and sternum, and in the hip (23) (Figure 1). These autoradiographs also show traces of mercury in the fetuses in a similar distribution to the maternal organs. Autoradiography of radioactive mercury is a good method to demonstrate mercury in organs, but it cannot detect the presence of mercury in individual cells.

Despite the suspicion that toxic metals such as mercury could contribute to the pathogenesis of connective tissue disorders, information is lacking as to whether cells in bone, joint, and fibrous tissues take up these metals selectively. One way of getting around the difficulty of measuring mercury in bones and joints is to study the early neonatal mouse, where the bones have not yet calcified. Using neonatal mice has the added advantage of allowing visualization of the whole body in transverse sections, so that connective tissues that are often not studied histologically in adults are included in the analysis. We therefore examined the cellular distribution of mercury in the tissues of neonatal mice that had been exposed to mercury vapor during gestation, using a histochemical technique, autometallography, that enables the detection of inorganic mercury within individual cells.

## Materials and Methods

### Mercury Exposure

Paraffin tissue blocks from a project that had studied the effect of prenatal mercury on the neonatal brain and spinal cord (24) were used to examine the distribution of mercury in the non-CNS organs of these neonates. Four pregnant C57 mice were exposed to 0.5 mg/m^3^ of mercury vapor in a mercury vapor chamber (25) for 4 h a day from gestational days 14–18, a non-toxic dose for rodents (26). One neonate from each litter was sacrificed with carbon dioxide on postnatal day 1 and immersed in 10% formalin for 48 h. Three mm thick transverse blocks of the body were processed routinely for paraffin embedding and microtomy. Negative controls were four neonatal mice that had not been exposed to prenatal mercury vapor.

### Autometallography

Seven micrometer sections of paraffin blocks were stained with silver nitrate autometallography to demonstrate inorganic mercury bound to sulfides and selenides, which is visible microscopically as black silver-coated grains (27). Briefly, sections were placed in physical developer containing 50% gum arabic, citrate buffer, hydroquinone and silver nitrate, covered with aluminum foil and placed in a water bath at 26°C for 80 min. Excess silver was removed by immersion in 5% sodium thiosulphate for 10 min and sections were counterstained with mercury-free hematoxylin. Adjacent sections were stained with hematoxylin only. For a mercury-positive control, a mouse spinal cord section, where motor neurons were known to contain mercury after an intraperitoneal injection of mercuric chloride (28), was included in each staining run.

### CD44 Immunostaining

Progenitor synovial and cartilage cells, and embryonic fibroblasts, express the phenotypic marker CD44 (29, 30). To see if mercury was present in these CD44-containing cells, sections were first stained with autometallography then treated with Epitope Retrieval Solution 1 at pH 6.0 for 20 min and immunostained with mouse anti-human CD44 antigen (Novocastra, UK) at 1:300, using a Bond III instrument (Leica). Bond Polymer Refine Red Detection (polymeric alkaline phosphatase-linker antibody conjugate) was used so that the black-staining autometallography deposits were not obscured by dark brown chromogens such as diaminobenzidine.

### Ethics

The methods to expose mice to mercury vapor, animal housing, handling, sacrifice, and tissue preparation had been approved by the University of Sydney Animal Ethics Committee (24). Because this work was undertaken on archived paraffin tissue blocks the ethics committee waived the requirement for renewal of the ethics protocol.

## Results

### Clinical

Mice from the same litters that had been prenatally exposed to mercury, but not sacrificed immediately after birth, suckled, and gained weight normally, and when followed for up to 40 days of age showed no abnormal features. The pregnant mice suffered no ill-effects from the mercury vapor exposure, either during or after pregnancy (24).

### Developing Joint and Bone

**Figure 2:**
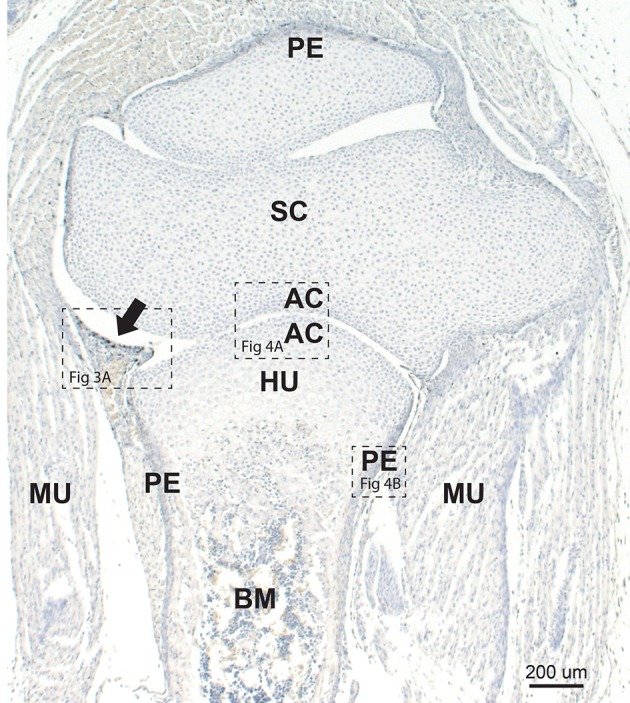
Neonatal mouse joint and bone. The developing synovium (arrow) projects into the synovial space between the scapula (SC) and humerus (HU). Mercury staining (i.e., black autometallography grains) is seen within the synovium (see higher magnification in Figure 3A). Chondrocytes adjacent to the joint space are in the region of the future articular cartilage (AC) (see higher magnification in Figure 4A). The periosteum (P) stains darkly due to the presence of mercury (see higher magnification in Figure 4B). Early bone marrow (BM) formation is seen. MU: striated muscle. Autometallography/hematoxylin.

In the large limb joints of neonatal mice, the developing synovium projects a short distance into the joint space, and often contains a large thin-walled blood vessel (Figure 2). Other nearby identifiable developing tissues are the articular chondrocytes, periosteum, bone marrow, and striated muscle. At this age mineralized bone has not yet formed.

### Autometallography

Mercury was seen on autometallography in the following tissues. The changes seen were similar in all neonatal mice that had been exposed to mercury prenatally.

#### Synovium

**Figure 3:**
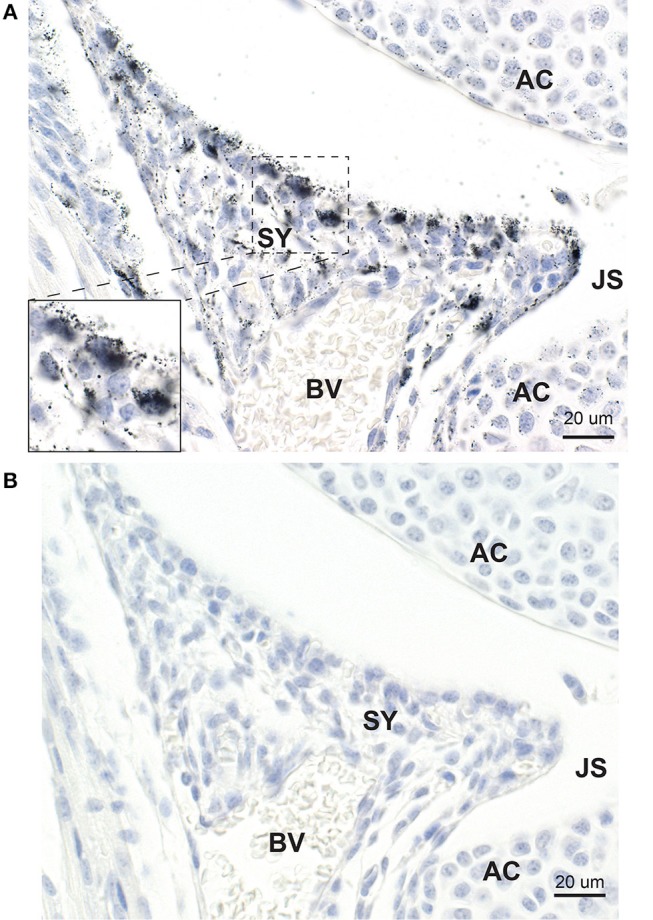
Mercury in synovial cells. **(A)** Mercury is present in a number of large and small synovial (SY) cells, predominantly those at the edge of the synovium adjacent to the joint space (JS). At higher magnification (inset) mercury is seen to occupy the cytoplasm of synovial cells. Nearby articular chondrocytes (AC) also contain mercury. BV: blood vessel. Autometallography/hematoxylin. **(B)** In an adjacent section not stained with autometallography, no black grains are seen in synovial cells or articular chondrocytes. Hematoxylin.

Mercury was present in many cells within the developing synovium, especially those situated at or near to the synovial surface (Figure 3A). None of the synovial cells that contained mercury stained with CD44. Adjacent sections stained with hematoxylin only showed no black grains in the synovium (Figure 3B).

#### Articular Chondrocytes

**Figure 4:**
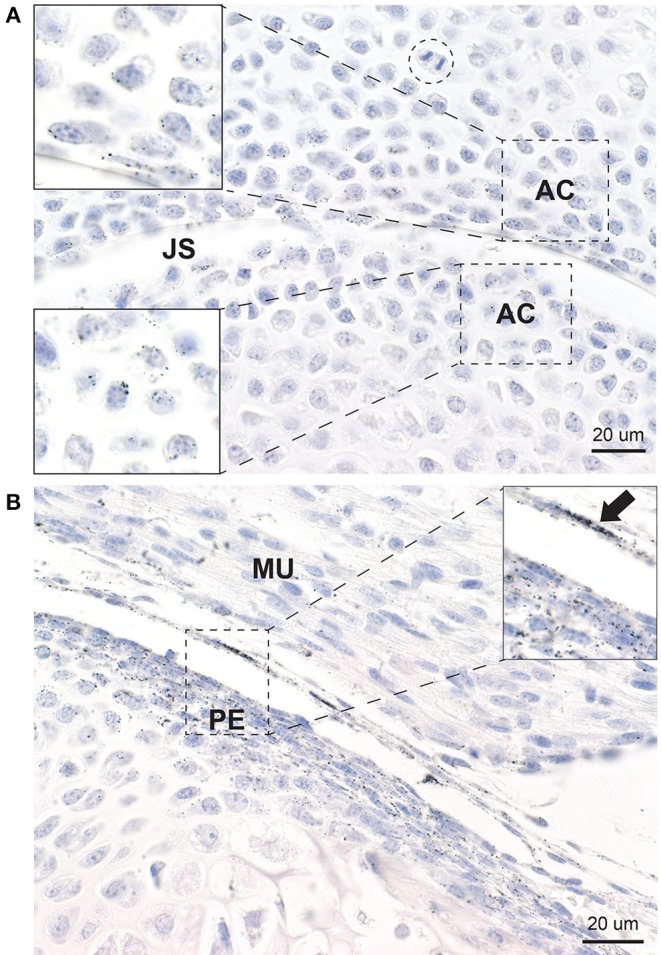
Mercury in articular chondrocytes and the periosteum. **(A)** Up to five rows of articular chondrocytes (AC) adjacent to the joint space (JS) contain mercury, but no mercury is seen in chondrocytes distant from the joint space. Mercury appears attached to the nuclear envelope of the articular chondrocytes (insets). A mitosis (dashed circle) is present. **(B)** Elongated periosteal cells (PE) and a thin membrane adjacent to the periosteum (arrow) stain positively for mercury (inset). MU: striated muscle. Autometallography/hematoxylin.

In all joints, mercury was present in the few layers of chondrocytes adjacent to the joint space (Figure 4A). Mercury appeared to attach preferentially to the nuclear envelope and plasma membrane of these chondrocytes. Chondrocytes distant from the joint space did not contain mercury.

#### Periosteum

Elongated periosteal cells of all developing long bones and vertebrae contained mercury, as did a thin membrane overlying the periosteum that was continuous with the synovium (Figure 4B). Deeper endochondral cells did not contain mercury.

#### Skin

**Figure 5:**
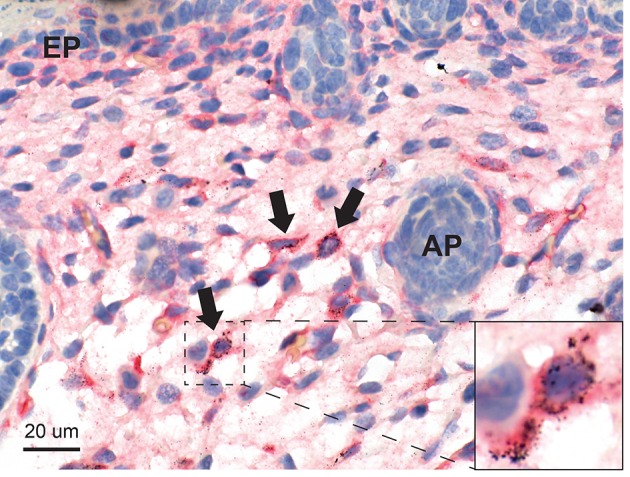
Mercury in the skin. Mercury in seen in scattered stellate and elongated fibroblasts of the deep dermis (arrows), some of which are CD44-positive (inset). Dermal appendages (AP) and the epidermis (EP) do not contain mercury. Autometallography/CD44/hematoxylin.

Mercury was present in elongated and stellate fibroblasts in the deep dermis (Figure 5) and in the subdermal connective tissue. Some of the fibroblasts containing mercury were CD44-positive. The dermal appendages and the epidermis did not contain mercury.

#### Blood Vessels

**Figure 6:**
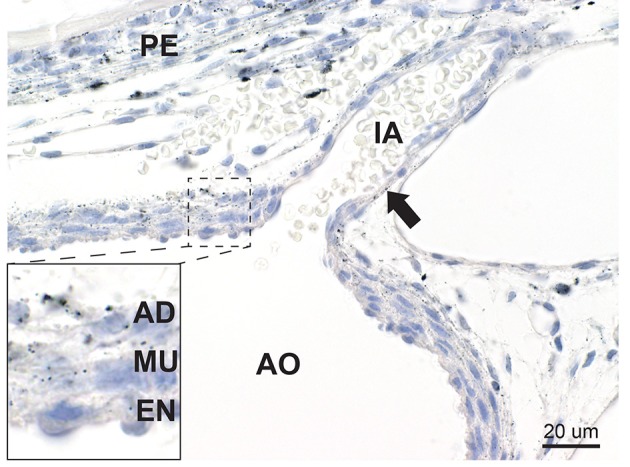
Mercury in blood vessels. In the aorta (AO) mercury is seen within elongated cells in the muscle layer (MU) and adventitia (AD) of the outer two-thirds of the posterior aortic wall, with smaller amounts in endothelial cells (EN) (inset). Mercury grains are also present in the endothelial cells of an intercostal artery (IA) (arrow). Mercury is seen in the periosteum (PE) of an adjacent vertebra. Autometallography/hematoxylin.

Mercury was present in elongated cells in the outer two-thirds (muscular and adventitial layers) of the posterior wall of the descending thoracic aorta (Figure 6), where it was close to the mercury-containing periosteum of the adjacent vertebrae. No mercury was seen in the abdominal aorta which was not near to the vertebrae. Mercury grains were also seen in the endothelial cells of smaller blood vessels, such as the intercostal arteries.

#### Trachea

**Figure 7:**
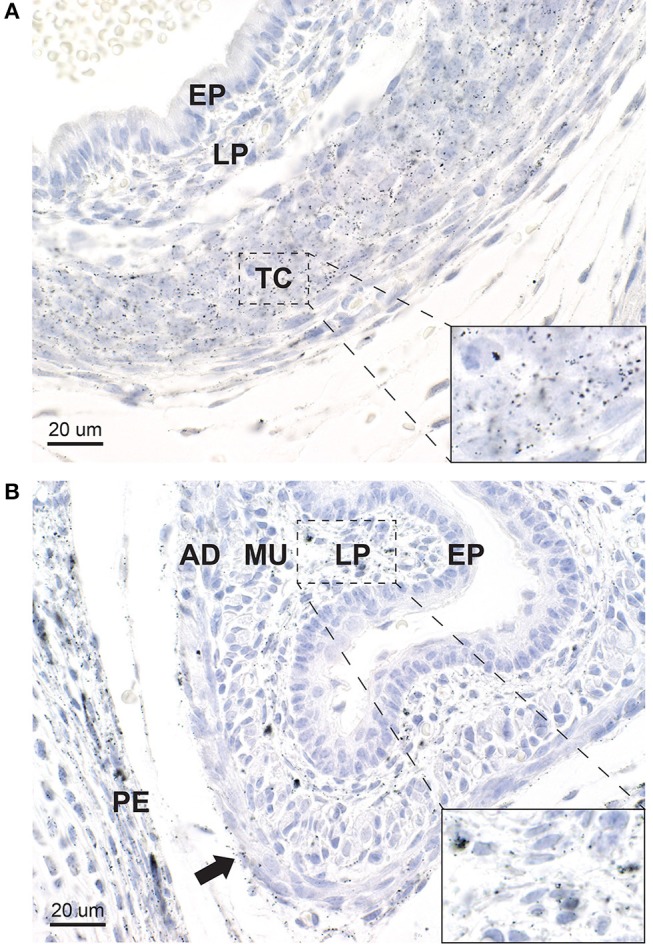
Mercury in the trachea and esophagus. **(A)** In the trachea, heavy mercury staining is seen in rounded chondrocytes and elongated fibroblasts in the tracheal cartilage (TC) (inset). A smaller amount of mercury is seen in elongated fibroblasts of the lamina propria (LP) underlying the tracheal epithelium (EP), which does not contain mercury. **(B)** In the esophagus, mercury is seen in many fibroblasts of the lamina propria (LP) (inset), and to a lesser degree in fibroblasts in the adventitia (AD) (arrow). No mercury is seen in the esophageal epithelium (E), but a small amount is present in the muscular layer (MU). Mercury is also present in the periosteum (PE) of an adjacent vertebra. Autometallography/hematoxylin.

A large amount of mercury was present within the trachea in the region of the future tracheal cartilage, both in chondrocytes and in elongated fibroblasts (Figure 7A). Smaller amounts of mercury were seen in fibroblasts of the lamina propria beneath the respiratory epithelium. The respiratory epithelium itself did not contain mercury.

#### Esophagus

Mercury was present in fibroblasts in the lamina propria beneath the esophageal epithelium, and in scattered cells of the adventitia (Figure 7B). The esophageal epithelium did not contain mercury.

#### Kidney

**Figure 8:**
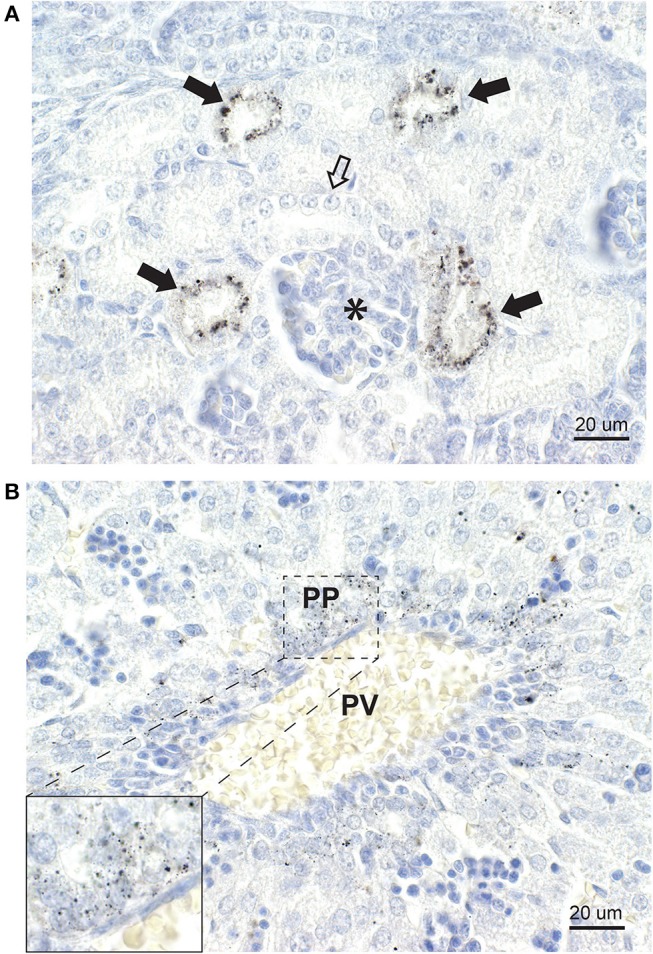
Mercury in the kidney and liver. **(A)** In the kidney, some cortical tubules stain strongly for mercury (closed arrows) while others (open arrow) contain no mercury. A glomerulus (asterisk) contains no mercury. **(B)** In the liver, a portal vein (PV) is surrounded by numerous periportal (PP) cells containing mercury (inset). No cells distant from the portal vein contain mercury. Autometallography/hematoxylin.

Mercury was present in the cells of renal cortical tubules (Figure 8A). No mercury was seen in the glomeruli or in the renal medulla.

#### Liver

Mercury was present in liver cells surrounding the portal veins (Figure 8B), but not in liver cells distant from the portal tracts.

#### Striated Muscle

Scattered elongated or stellate mercury-containing fibroblasts, some CD44-positive, were present in striated muscle between myofibers. Myofibers themselves did not contain mercury.

#### Other Organs

No mercury was seen in the heart, lungs, pancreas, spleen, or intestines, but mercury was present in capillary endothelial cells of the brain and spinal cord.

### Control Neonates

Neonatal mice that had not been exposed to prenatal mercury had no autometallography staining in any tissues.

## Discussion

Key findings in this study are that mercury localizes to the synovial cells and articular chondrocytes that are affected in two major joint disorders, rheumatoid arthritis and osteoarthritis. In addition, mercury is retained postnatally in connective tissue fibroblasts in several organs, many of which are involved in multi-organ connective tissue disorders. Mercury appears to attach to the nuclear membrane of cells, a feature noted previously in electron microscopic localization of mercury within cells (31), which could be pertinent to the common finding of anti-nuclear antibodies in connective tissue disorders (17–20). While these findings do not provide direct evidence that mercury is involved in the pathogenesis of connective tissue disorders, the finding of mercury within the cells most affected by these disorders suggests that further experiments to test the hypothesis that toxic metals play a part in these disorders are warranted.

Rodents in late gestation develop a chorioallantoic placenta similar to that of humans (32), and pregnant women are commonly exposed to mercury due to fish consumption or mercury-containing amalgam dental restorations (33). There is therefore reason to believe our findings in the mouse could be applicable to mercury uptake in humans. Mercury affects the epigenetic profile of cells (34) and damages DNA (35), so finding mercury in this range of connective tissue cells has implications for susceptibility to a number of later-life disorders that effect the synovium, cartilage, and bone. Although we studied the fetal uptake of mercury, autoradiographic studies indicate that mercury is also taken up and retained by adult vertebrate bone and joint (23). This implies that mercury uptake is not confined to developing cells but also occurs in adults. This fits with our finding that most of the cells in our study taking up mercury did not contain CD44 and so are likely to be mature cells.

The reason this assortment of cells in our mice has a propensity to take up mercury is unclear. However, a pointer may come from our kidney findings, where some renal tubules stained strongly for mercury. Mercury transporters have been identified in a number of tissues, but the best defined are those in the proximal renal tubule (36). The finding of mercury in non-renal cells in our mice, in the presence of renal tubule mercury uptake at the same exposure time and mercury dose, suggests that mercury transporters may also be present in the synovial, cartilage, periosteal, fibroblastic, and endothelial cells in which mercury was seen.

A major pathological feature of rheumatoid arthritis is a persistent synovitis with altered behavior in fibroblast-like synovial cells (37), and the synovium was a conspicuous target of mercury uptake in our mice. Although about 50% of the risk of developing rheumatoid arthritis is attributed to genetic variants, environmental factors remain largely unknown, apart from an increased risk from smoking (37). The reason for the link between smoking and rheumatoid arthritis remains unknown, though factors such as oxidative stress, inflammation, epigenetic changes and antibody formation have been proposed (38). At first glance it seem unlikely that mercury is responsible for this link since only a small amount of mercury is present in cigarette smoke (39). However, the large amount of cadmium in cigarette smoke (40) may be relevant, since cadmium exposure is a proposed risk factor for rheumatoid arthritis (41), and synergistic actions between toxic metals such as cadmium and mercury are recognized (42). Cadmium and mercury from cigarette smoke could therefore accentuate each other's toxicity within the synovium.

The autoantibodies in rheumatoid arthritis against IgG (rheumatoid factor) and citrullinated peptides suggest rheumatoid arthritis is an autoimmune disorder (4, 43), and the propensity for mercury to trigger autoimmunity is now established (13, 14, 17, 44). Of relevance regarding human exposure to mercury, studies in Brazil of artisanal gold miners (who use mercury to extract gold) showed these miners have a high prevalence and higher titers of antinuclear and antinucleolar antibodies, higher concentrations of serum pro-inflammatory cytokines (45, 46), and have an array of other autoantibodies as well (47). A subset of people consuming mercury-affected fish in Brazil had raised antinuclear antibodies and changes in cytokine profile, suggesting a specific phenotype of mercury susceptibility (48). These findings indicate that mercury exposure can lead to autoimmune dysfunction and systemic inflammation in humans.

Articular cartilage and synovium appear to play key roles in the pathogenesis of osteoarthritis (49–51), and both these tissues took up mercury in our mice. The immune system is activated in osteoarthritis (50), and epigenetic variation has been implicated in this disease (52), both mechanisms in which mercury plays a part (15). One puzzling feature of osteoarthritis is why obesity should be a risk factor for the disease in non-weight bearing joints (50). Raised blood levels of mercury have been found in people with obesity (53, 54) and a link has been made between adipokines arising from fatty tissue (55, 56) and osteoarthritis (57). Mercury raises the levels of damaging adipokines from fatty tissue (58), so mercury may be one factor underlying the co-occurrence of obesity and osteoarthritis.

A severe early-onset form of osteoarthritis, Kashin-Beck disease, is found in parts of China where soil selenium levels are low (59). Dietary selenium is a protective factor against mercury toxicity (60) so a lack of selenium in regions affected by Kashin-Beck disease may allow mercury to damage articular cartilage and synovium, with consequent early-onset osteoarthritis.

Cartilage destruction is a prominent feature in both osteoarthritis and active rheumatoid arthritis (61), and it has been proposed that cartilage damage in these disorders is mediated by matrix metalloproteinases (62–64). Of relevance to our findings, mercury exposure increases circulating levels of some matrix metalloproteinases (65), and certain polymorphisms in matrix metalloproteinase-9 lead to increased levels of the proteinase in people exposed to low doses of environmental mercury (66). It would therefore be of interest to see whether people with rheumatoid arthritis or osteoarthritis have higher than expected circulating levels of metalloproteinase-9 levels if they have the identified polymorphisms combined with low levels of circulating mercury.

A striking finding in our neonatal mice was the amount of mercury that was taken up by developing periosteal cells. The periosteum contains osteogenic cells that help form and repair cortical bone (67, 68) and attempts have been made to increase periosteal osteogenic activity to treat osteoporosis (69). The causes of osteoporosis remain largely unknown (70), though environmental factors are considered to play a part (71). One such environmental factor could be mercury taken up by periosteal cells, since mercury affects the epigenome (16), which appears to be involved in osteoporosis (72).

Chondrocytes and fibroblasts in the trachea of our neonatal mice took up mercury avidly. If the same mercury uptake occurs in humans, this could affect the postnatal formation of cartilage within the trachea, so prenatal mercury exposure could an environmental factor underlying the collapsibility of the trachea found in infants with tracheomalacia (73). Furthermore, given the ability of mercury to trigger inflammation (14), episodic exposure to mercury with widespread uptake of mercury in cartilage could trigger the condition of relapsing polychondritis (74).

A puzzling finding was that mercury was found in the aorta only in its posterior wall. This may relate to the proximity of the posterior wall of the aorta to the anterior aspect of the vertebral bodies, whose periosteum contained large amounts of mercury in our mice. Atherosclerosis has been noted to be more prevalent in people with rheumatoid arthritis than in the general population (75), though the cause behind this association is not known. The posterior wall of the aorta is the region first affected by atherosclerosis (76), which may be of relevance since mercury is associated with the risk of atherosclerosis (77) and the metal is known to affect the function of endothelial cells (78). Our findings therefore raise the possibility that the increased atherosclerosis seen in rheumatoid arthritis could be due to underlying mercury toxicity in both synovial and endothelial cells.

The wide range of cells containing mercury in our mice fits with the finding that multiple tissues are often involved in the connective tissue disorders. For example, in rheumatoid arthritis, although the primary damage appears to be the joint synovium, several other organs and tissues including skin, blood vessels, cartilage, esophagus, kidney, and liver are often damaged (79, 80), and all of these tissues in our mice contained mercury. However, not all the tissues in our mice that are commonly affected by rheumatoid arthritis, such as the lung parenchyma, the pericardium and the pleura (81) contained mercury, so other factors may be operating in these organs. Apart from rheumatoid arthritis, the widespread uptake of mercury in connective tissues could be responsible for the multiple organ involvement of people who suffer from systemic sclerosis (10, 12, 18), systemic lupus erythematosus (8), mixed connective tissue disease, polymyalgia rheumatica and fibromyalgia (11).

This study has a number of limitations. (**1)** This is mouse model, but the cellular distribution of mercury in human bone, joint, and connective tissues remains unknown. In addition, only limited information is available on the cellular distribution of mercury in non-connective tissues of humans known to have been exposed to mercury. In one man who injected himself with metallic mercury, mercury deposits were found in renal tubules and periportal hepatocytes (82), similar to our mouse mercury uptake. Therefore, at least some similarity of mercury tissue distribution in mice and humans is likely. However, differences in mercury distribution have also been noted between these two species, for example in the uptake of mercury by certain cells in the brain (83). At present, no techniques are available to explore the cellular distribution of mercury in the tissues of living humans, but future imaging techniques may be able to achieve this. (**2**) In this experiment only the vapor form of mercury was used, which is relevant to humans in certain occupations and those with amalgam fillings (6). However, humans are often exposed to methylmercury from eating larger mercury-containing fish, so further mouse experiments using organic mercury exposure are needed to see if this form of mercury also targets connective tissues. (**3)** Disorders such as rheumatoid arthritis and osteoarthritis are likely to result from gene-environment interactions (50, 84), but we studied only one environmental toxicant, mercury. To obtain more evidence that toxic metals could trigger connective tissue diseases, further studies using this toxicant model could be combined with strains of mice that are genetically susceptible to develop an autoimmune reaction to mercury (21), with mice exposed to cigarette smoke (38), or with mice on a low selenium diet which could potentiate mercury toxicity (85). In addition, other metal toxicants such as lead (86) and cadmium (41) could be studied for their effects on connective tissues. In the case of osteoarthritis, this toxicant model could be used to see if osteoarthritis develops earlier than expected in genetic strains of mice who develop spontaneous osteoarthritis or obesity, or in mice who have a destabilized medial meniscus to predispose them to osteoarthritis (51).

In conclusion, mercury is taken up selectively by mouse synovium and articular chondrocytes, tissues that are affected in rheumatoid arthritis and osteoarthritis, as well as by fibroblasts, periosteal cells and endothelial cells. Mercury provokes autoimmune, inflammatory, and genetic and epigenetic changes that have been described in joint and connective tissue diseases. Our findings lend weight to the hypothesis that mercury could trigger these disorders in people with genetic or epigenetic susceptibilities that promote autoimmunity, inflammation, mercury transport into cells, or mercury-induced cellular toxicity. Further experiments combining a variety of toxicant exposures, with known genetic and environmental susceptibilities to a range of connective tissue disorders, will enable more light to be cast on the role of toxic metals in these diseases.

## Data Availability

All datasets generated for this study are included in the manuscript and/or the supplementary files.

## Ethics Statement

The methods to expose mice to mercury vapor, animal housing, handling, sacrifice, and tissue preparation had been approved for a previously published study on mercury uptake in the brain by the University of Sydney Animal Ethics Committee. Because this present work was undertaken on archived paraffin tissue blocks the ethics committee waived the requirement for renewal of the ethics protocol.

## Author Contributions

RP conceived and planned the study, analyzed the data, and drafted the manuscript. RP and SK performed the experiments. Both authors revised and approved the article.

### Conflict of Interest Statement

The authors declare that the research was conducted in the absence of any commercial or financial relationships that could be construed as a potential conflict of interest.
